# Bilateral Simultaneous Full-Thickness Macular Holes: A Case Report with Spontaneous Resolution

**DOI:** 10.3390/reports8020040

**Published:** 2025-03-28

**Authors:** Isabel López-Bernal, Ángel Sánchez Trancón, Pedro Serra

**Affiliations:** Ophthalmology Clinic Vista Sánchez Trancón, 06010 Badajoz, Spain

**Keywords:** macular hole, spontaneous closure, bilateral, optical coherence tomography

## Abstract

**Background and Clinical Significance**: Full-thickness macular hole (FTMH) is a common retinal condition that impairs detailed vision, with idiopathic causes being the most prevalent. Small macular holes (<250 µm) have the potential for spontaneous closure, whereas larger holes typically require surgical intervention to restore visual acuity (VA). The management of small macular holes remains controversial, as approximately 25% resolve spontaneously. Reporting cases of spontaneous closure may provide evidence to support a conservative, non-surgical approach in such cases. **Case Presentation**: We report the case of a 65-year-old female patient monitored using spectral domain optical coherence tomography (OCT). She initially presented with unilateral FTMH and subsequently developed an FTMH in the fellow eye during follow-up. Both macular holes closed spontaneously, leading to VA recovery. OCT imaging enabled the identification of vitreomacular traction as the underlying mechanism for hole formation and the bridging process responsible for spontaneous closure. **Conclusions**: This case highlights the potential for spontaneous anatomical and functional recovery in small-diameter macular holes (<250 µm). A conservative, observational approach may be appropriate in selected cases, potentially avoiding unnecessary surgical intervention.

## 1. Introduction and Clinical Significance

Macular hole (MH) is a full-thickness anatomical defect located in the fovea, the central area of the retina responsible for detailed vision. This defect often leads to a significant reduction in visual acuity (VA) [1]. The incidence of MH is estimated to range from 4.1 to 7.8 cases per 100,000 individuals annually, with approximately 87% of cases classified as idiopathic [2,3]. Other etiologies include trauma and ocular comorbidities, such as high myopia, cystoid macular edema, and neovascular age-related macular degeneration [4].

The pathogenesis of idiopathic macular holes is primarily linked to vitreomacular traction caused by the abnormal adhesion of vitreous cortex fibers to the internal limiting membrane (ILM). The vitreous cortex, which remains attached to the vitreous base, is in close contact with the macular–foveal region. With normal aging, vitreous liquefaction induces contraction of the vitreous body, creating anterior–posterior traction in the macular region. Furthermore, vitreous dynamics during ocular movements generate tangential traction, which may also contribute to MH formation [4]. Histologically, the pathogenic process progresses through distinct stages, ranging from the spontaneous resolution of a previous MH in one eye without foveal architecture change and vitreomacular adhesion in the fellow eye (Stage 0) to more advanced stages, i.e., MH with outer retinal elevation from the retinal pigmentary epithelium (RPE) at the foveal center (Stage 1), full-thickness macular hole (FTMH) with a horizontal diameter ≤400 µm and vitreomacular adhesion (Stage 2), FTMH with a horizontal diameter >400 µm without vitreomacular adhesion (Stage 3), and FTMH characterized by disruption of the retinal layers and complete vitreous detachment (Stage 4) [1].

The International Vitreomacular Traction Study (IVMTS) introduced a refined classification system based on optical coherence tomography (OCT) imaging. This classification system enables the precise assessment of the retinal profile, particularly the minimal horizontal macular diameter opening. FTMHs are categorized as small, medium, or large, based on measurements of <250 µm, ≥250 µm and <400 µm, and ≥400 µm, respectively [5].

Surgical intervention is the recommended treatment for visual recovery in Stage 2 MH (FTMH <400 µm with vitreomacular adhesion) and more advanced stages [6]. Nevertheless, studies report spontaneous closure rates for FTMH ranging from 2.9% to 12.3% for Stages 2 to 4, with rates as high as 22% for small FTMHs (<250 µm) [7]. Recently, a retrospective study examining the timeline for spontaneous closure of idiopathic FTMH found a mean closure time of 44 days after diagnosis, with the closure time being positively correlated with MH horizontal diameter [8]. Notably, spontaneous closures are associated with VA outcomes comparable to those achieved through surgical procedures [9,10,11]. Bilateral FTMH is estimated to occur in approximately 10% of cases, typically within 48 months of the initial eye developing the condition [12]. The occurrence of simultaneous bilateral holes with spontaneous resolution is rare, with only a few case reports detailing the pathology’s evolution [13,14,15].

This report presents a rare case of simultaneous bilateral FTMH, highlighting MH development in one eye and posterior spontaneous resolution in both eyes.

## 2. Case Presentation

**Figure 1:**
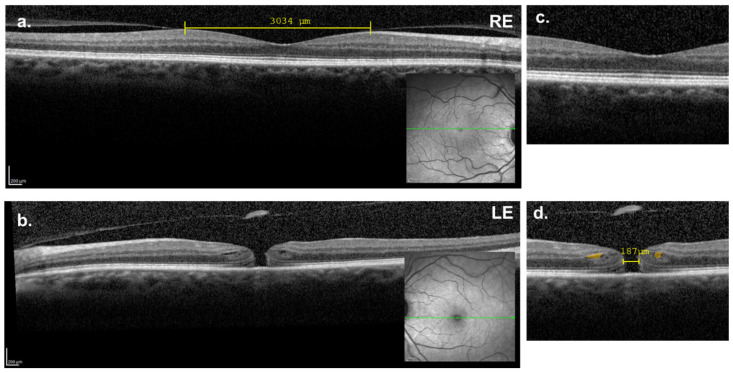
(**a**,**c**) Macular OCT showing a vitreomacular adhesion in the right eye. The posterior hyaloid is detached nasally and temporally to the fovea. (**b**,**d**) Macular OCT of the left eye showing a full-thickness macular hole (horizontal diameter = 187 μm) with intraretinal cystic spaces (colored in orange), as well as detachment of the posterior hyaloid with operculum adherence. (**c**,**d**) Details of the foveal region.

In July 2020, a 65-year-old Caucasian female presented for an ophthalmologic examination with symptoms of reduced VA and a central cloudy sensation in her left eye (LE). On examination, the patient exhibited mild hyperopia in both eyes: right eye (RE) +4.00 D and LE +4.00 D. The best-corrected visual acuity (BCVA) was 20/22 in the RE and 20/67 in the LE. Intraocular pressure was 16 mmHg in both eyes measured by Goldmann tonometry. A dilated slit-lamp examination revealed early-stage cataracts in both eyes. Indirect ophthalmoscopy identified an FTMH in the LE. Macular OCT (Heidelberg Spectralis, Heidelberg, Germany) of the RE revealed vitreomacular adhesion, with horizontal adherence points measuring 3039 µm apart, and normal foveal anatomy (Figure 1a). In the LE, OCT imaging showed an FTMH with a horizontal diameter of 208 µm (Stage 2), with exposure of the RPE, accompanied by small cystic spaces mainly in the outer plexiform layer (OPL) and the Henle fiber layer (HFL), as well as curling of the photoreceptors layer and external limiting membrane (ELM). In the vitreous cavity, detachment of the posterior hyaloid was observed, as well as an attached operculum resulting from the retinal hole (Figure 1b).

**Figure 2:**
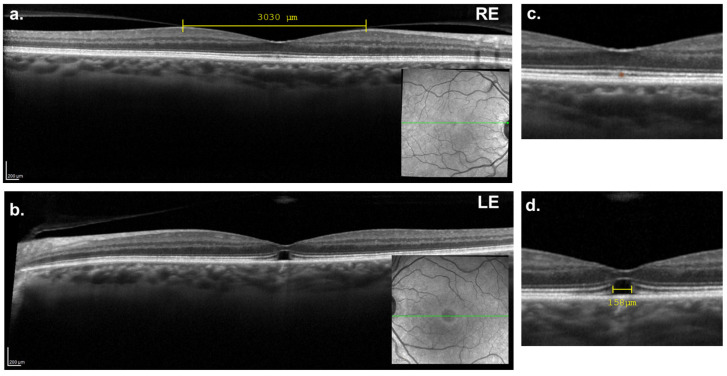
Two-months follow-up macular OCT. (**a**,**c**) Right eye showing a vitreomacular adhesion, with the presence of small cyst in the photoreceptors outer layer, colored in orange (**c**). (**b**,**d**) Left eye showing a partial closure of the macular hole. The “bridging effect” is characterized by the restructuration of the retina internal layers. A partial macular hole persisted in the more external retinal layers (MH horizontal diameter = 158 μm).

The options of surgical treatment and observational follow-up were discussed with the patient. Due to time constraints related to her relocation abroad, she opted for observational follow-up. Upon her return two months later, partial spontaneous closure of the LE macular hole was observed, with BCVA improving to 20/30. The RE retained a BCVA of 20/22, with OCT imaging showing persistent vitreomacular adhesion and the appearance of a small cyst in the outer segment of the photoreceptors (Figure 2a,c). OCT imaging of the LE revealed the formation of bridging tissue between the inner retinal layers, extending from the internal limiting membrane (ILM) to the ELM, although disruption of the photoreceptors layers persisted (Figure 2b,d).

**Figure 3:**
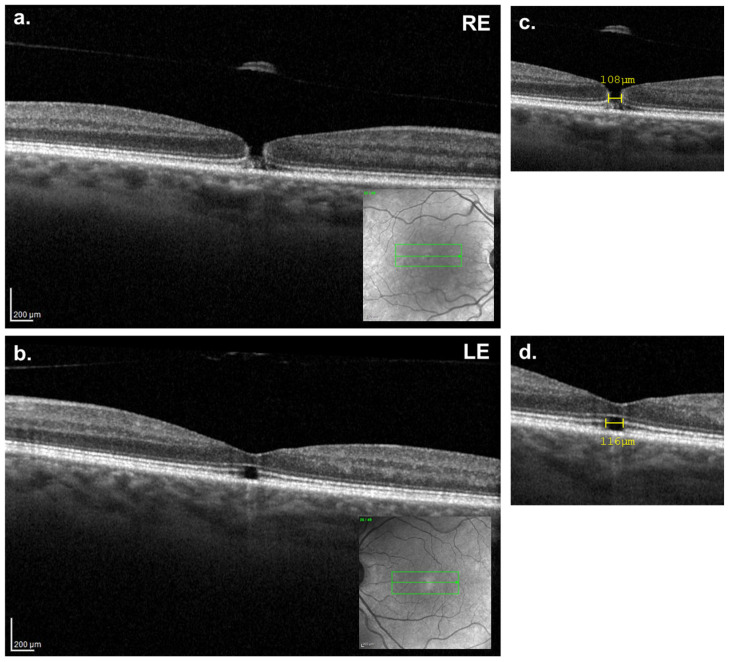
Six-months follow-up macular OCT. (**a**,**c**) Right eye showing a full-thickness macular hole (minimal horizontal size = 108 μm) with an operculum adhered to the posterior hyaloid. (**b**,**d**) Left eye continuing to show a partial closure of the macular hole with a reduction of the MH horizontal diameter to 116 μm.

At the six-month follow-up, the patient reported reduced vision in the RE (VA: 20/30), while the LE remained stable at VA 20/30. OCT imaging of the RE revealed the development of an FTMH with a horizontal diameter of 107 µm, clearly visible between the ILM and the ELM, along with complete detachment of the posterior hyaloid and operculum adherence (Figure 3a,c). The inner–outer photoreceptors segment line (IS-OS line or ellipsoid zone) was minimally preserved, with dehiscence of the photoreceptors primarily in the outer-segment photoreceptors layer. In the LE, the defect in the outer retinal layers persisted (Figure 3b,d), but the MH horizontal diameter was reduced to 116 μm.

**Figure 4:**
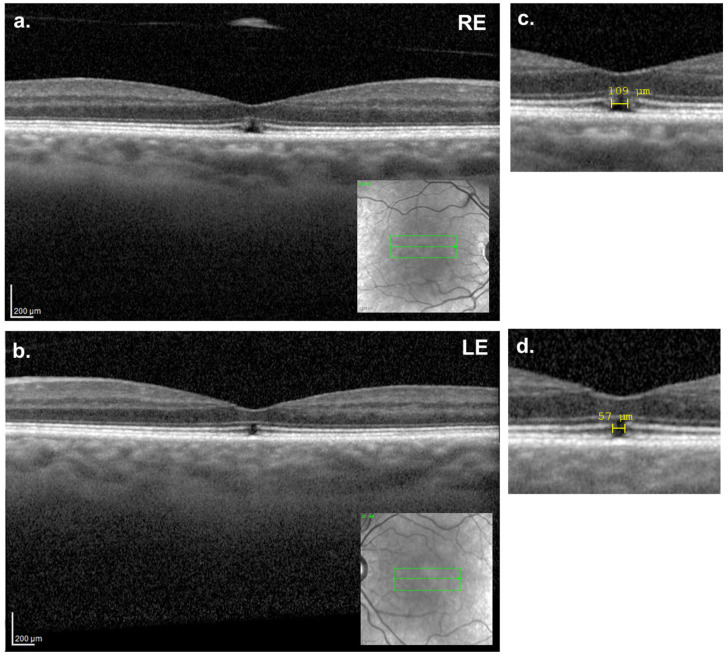
Seven-months follow-up macular OCT. (**a**,**c**) Right eye showing the partial closure of the inner retinal layers, with the remaining outer retinal containing a small lamellar hole (109 µm). (**b**,**d**) Left eye showing a minimal defect (57 µm) on the outer retinal layers. In the detail images (**c**,**d**), there is still a disruption of the photoreceptors’ layers.

Given the small size of the RE macular hole and the favorable evolution observed in the LE, a conservative expectant approach was agreed upon with the patient. At the seven-month follow-up (Figure 4a–d), partial closure of the MH in both eyes was observed, with the persistence of small lamellar MHs in the outer retinal layers (horizontal diameter: RE = 109 μm; LE = 57 μm). There was no significant improvement in BCVA, which remained stable at 20/30 in both eyes.

**Figure 5:**
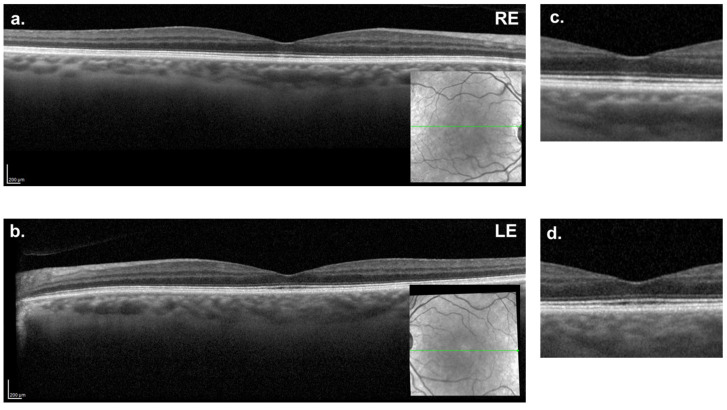
Twenty-three months follow-up macular OCT. (**a**–**d**) Right eye and left eye showing full recovery of the anatomical structure, respectively.

Twenty-three months after the initial presentation, complete anatomical closure of the MH was confirmed in both eyes (Figure 5a–d), with the continuity of the IS-OS line junction restored in both eyes. However, despite the anatomical improvement, the BCVA remained stable at 20/30 in both eyes.

## 3. Discussion

This case report describes a rare instance of bilateral spontaneous FTMH closure, contributing to the growing evidence supporting a more conservative therapeutic approach for small FTMH as an alternative to surgical intervention [13]. Compared to previous case reports [13,14,15], this case further illustrates the role of vitreomacular traction in MH development and emphasizes the importance of considering risk signs in managing the pathology. The LE presented with an FTMH and an operculum attached to the posterior hyaloid, suggesting that a previous vitreous adhesion and tractions were responsible for the MH formation [4]. Notably, six-months after the initial presentation, the RE developed an FTMH, where a clear vitreomacular adhesion had been visible on previous OCT scans. Bringman et al. [16] proposed that the anterior vitreofoveal traction exerted by the posterior hyaloid, when attached to the foveola, induces the detachment of the inner Müller cell layer from the outer nuclear layer (ONL). This process leads to the elongation of the Müller cell cone stalk and the subsequent formation of a pseudocyst in the foveola. Persistent vitreofoveal traction ultimately results in the detachment of the posterior hyaloid, accompanied by adherent Müller glial tissue (operculum), as observed bilaterally in the present case. The loss of the Müller cell cone stalk exposes the foveal walls to centrifugal and anterior forces, influenced by the oblique orientation of Müller cells. Consequently, this structural disruption leads to ONL dehiscence, increased foveal wall curvature, and the formation of an FTMH.

Previous studies on the bilaterality of macular holes report an incidence of secondary MH in the fellow eye ranging from 9.3% to 13% over an approximately four-year period [12,17]. A recent review reported an incidence of bilateral macular holes between 0% and 7% at presentation and between 0% and 28% during a follow-up period ranging from 19 to 57 months [7]. Identified risk factors for the development of a second-eye MH include vitreoretinal traction, inner foveal cysts, and outer foveal defects detected on OCT [17]. At presentation, the patient exhibited a Stage 2 FTMH in the LE and vitreomacular adhesion in the RE, placing her at increased risk of developing an MH in the fellow eye. An FTMH in the RE developed approximately six months after appearing in the LE, a shorter fellow-eye onset period compared to that in the literature. Female gender and an age between the sixth and seventh decade seem to be risk factors for idiopathic FTMH; however, their influence on the pathogenesis of MH has yet not been clearly identified [18].

Pars plana vitrectomy remains the standard treatment for MHs classified as Stage 2 and higher [9]. However, substantial evidence suggests that spontaneous resolution is possible for Stage 2 MHs, as classified by Gass, particularly those ≤250 µm in size according to the IVTS staging system. A recent review found that spontaneous closure rates for FTMH ≤250 µm are approximately 22%, with rates decreasing for larger or more advanced holes [7]. Another recent retrospective study reported a spontaneous closure rate of 18% in a population with MHs smaller than 200 µm [10]. Given that the LE’s FTMH had a horizontal diameter of 208 µm and no vitreomacular adhesion, a conservative observational approach was chosen, involving close monitoring via OCT imaging. OCT imaging provided critical insights into changes in the MH size. For instance, in the LE. the horizontal diameter between MH walls was 187 µm at presentation, reducing to 158 µm at two-month follow-up, 116 µm at six-months follow-up, and 57 µm at seven-months follow-up, confirming progressive MH reduction and enabling the detailed monitoring of the closure mechanism.

The most reported mechanism of spontaneous FTMH closure is retinal tissue bridging, in which retinal cell proliferation across the hole facilitates closure from the inner to the outer retinal layers. This mechanism prevents the influx of vitreous fluid into the retinal spaces, allowing for the restoration of the retinal layers [7]. According to Brigmann et al. [16], the restoration of retinal layers occurs through the fusion of the remaining Müller cell cone and Müller cell structures at the OPL and the inner part of the ONL. This process is facilitated by the centripetal contraction of Müller cell-side processes within the OPL. Normal foveal architecture is restored as the central ONL thickens, driven by the centripetal migration of the photoreceptor cell somata, assisted by Müller cells, towards the foveal centre. The time required for complete MH closure correlates with hole size; smaller MHs (≤250 µm) tend to close within an average of 3.3 months, ranging from 0.75 to 11 months, whereas larger holes (>250 to 400 µm) close in approximately 7 months (a range of 1–24 months) [7]. In the LE, near-complete closure was observed eight months post-presentation, and the RE exhibited a shorter recovery time (1 to 2 months), likely due to its smaller MH size [8]. However, the full anatomical retina was only observed nearly two years after LE presentation.

In terms of functional recovery, the LE showed an improvement in BCVA from 20/67 to 20/30, consistent with the literature [7,8,10]. In contrast, the RE demonstrated no significant gain in BCVA, although its VA at the onset of the Stage 2 FTMH was comparable to the LE’s VA after complete MH closure. OCT imaging revealed an apparent recovery of the IS-OS junction approximately two years post-onset, a fundamental factor for functional recovery [16,19]. This aligns with findings from long-term studies reporting complete macular microstructural recovery within three years [20].

It is important to note that cataract progression during the two-year follow-up may have contributed to the final BCVA in both eyes. This underscores the need to consider other ocular comorbidities that may limit visual recovery, even after the successful anatomical closure of macular holes [21].

## 4. Conclusions

Careful evaluation and continuous follow-up are essential for identifying patients who may benefit from a conservative management approach. Regular follow-up with detailed OCT imaging and precise functional assessments is crucial for determining whether an expectant approach (spontaneous MH closure) or surgically based approach (vitrectomy) is most appropriate for achieving optimal VA recovery. Understanding the mechanisms underlying spontaneous MH closure may facilitate the development of less-invasive therapeutic strategies, reducing the risks associated with conventional surgery, and broadening treatment options for a wider patient population. This case highlights the importance of an individualized approach to macular hole management, emphasizing the need to consider the anatomical characteristics and the patient’s unique clinical context to optimize outcomes and enhance quality of life.

## Data Availability

The data used for this study must be requested from the corresponding author due to privacy concerns.

## Abbreviations

The following abbreviations are used in this manuscript:

BCVA	Best-corrected visual acuity
ELM	External limiting membrane
FTMH	Full-thickness macular hole
HFL	Henle fiber layer
INL	Internal limiting membrane
IVMTS	International vitreomacular traction study
LE	Left eye
MH	Macular hole
OCT	Optical coherence tomography
ONL	Outer nuclear layer
OPL	Outer plexiform layer
RE	Right eye
VA	Visual acuity
